# Evaluation of double-high insert mid-term outcomes in cruciate-retaining medial-pivotal total knee arthroplasty – a propensity score-matched analysis with averaged 8-year follow-up

**DOI:** 10.1186/s12891-022-05484-6

**Published:** 2022-06-14

**Authors:** Wenzhe Wang, Shuai Xiang, Yingzhen Wang, Chengyu Lv, Changyao Wang, Haining Zhang

**Affiliations:** Department of Joint Surgery, the Affiliated Hospital of Qingdao University, Qingdao, 266000 Shandong China

**Keywords:** Total knee arthroplasty, Medial-pivotal, Double-high, Cruciate-retaining

## Abstract

**Background:**

This study aimed to compare the mid-term clinical and radiographic outcomes between medial-pivotal (MP) insert and double-high (DH) insert used under the cruciate-retaining condition in ADVANCE® total knee arthroplasty (TKA).

**Methods:**

The follow-up was conducted for 158 consecutive patients who underwent unilateral ADVANCE® TKA from January 2011 to April 2014. Eighty-four MP inserts and 74 DH inserts were used under cruciate-retaining conditions. A 1:1 propensity score matching (PSM) analysis was performed between MP inserts and DH inserts to compare the clinical and radiographic outcomes.

**Results:**

After a 1:1 PSM, 120 patients (60 pairs) were matched between the MP and DH inserts groups. The baseline demographic parameters and clinical scores were comparable between the two groups. The postoperative clinical outcomes at an averaged 8-year follow-up of both groups were significantly improved. The range of motion (ROM) of the DH group was better than that of the MP group, and equivalent Knee Society Function Score (KSFS) between the two groups was found. However, the Knee Society Score (KSS), Western Ontario and McMaster Universities Arthritis Index (WOMAC) score, and Forgotten Joint Score (FJS) of the MP group were found to be significantly superior to those of the DH group. Comparable complication and revision rates were observed between the two groups. The radiographic results were also equally good between MP and DH groups.

**Conclusions:**

Although the mid-term clinical and radiographic outcomes of the DH inserts are fairly good, the clinical scores of the DH group were worse than those of the MP group.

## Introduction

Total knee arthroplasty (TKA) is the most successful procedure for patients with end-stage osteoarthritis and good to excellent long-term functional outcomes, and implant survivorship has been validated [1–4]. However, the post-cam design in conventional knee implants fails to fully restore the typical motion pattern of the knee, leading to a “paradoxical anterior slide” of the femur during knee flexion, which has been considered to contribute to patients’ dissatisfaction [5]. Physiologically, the medial-pivotal motion and femoral rollback are observed throughout the flexion motion, even in a deep flexion range [6]. To achieve better function, the medial-pivotal (MP) concept has been emphasized during implant design.

ADVANCE® knee (Wright Medical Technology, Arlington, TN) is one of the earliest MP-designed implants and has been widely used in the past decade, presenting excellent clinical outcomes and survivorship [7, 8]. During knee flexion, the medial ball-in-socket structure of the standard MP insert provides anterior-posterior stability instead of the post-cam system. Meanwhile, the arcuate groove of the lateral side allows the femoral rollback centered on the medial axis. With a congruent articular surface, the normal medial-pivotal kinematics is better restored, and several studies have demonstrated decreased intercondylar stress and lessened polyethylene wear in this implant [9–12]. A double-high (DH) insert, which means “high stability, highflexion”, is an alternative during clinical practice despite the standard MP insert. The posterior lip of the DH insert is 3 mm lower than that of the standard MP insert, resulting in a posterior slope to facilitate the femoral rollback during knee flexion [13].

But at present, there is little research on DH. In order to compare the mid-term clinical and radiographic outcomes between MP and DH inserts in cruciate-retaining ADVANCE® TKA, we conducted an average 8-year follow-up.

## Materials and methods

This study was approved by the review board of the Affiliated Hospital of Qingdao University (QYFY QZLL 26921). The medical records of consecutive patients who underwent unilateral cruciate-retaining ADVANCE® (Wright Medical Technology, Arlington, TN) TKAs from January 2011 to June 2014 due to osteoarthritis were reviewed.

The standard of inclusion criteria: (1) primary total knee arthroplasty. (2) No previous knee surgery history before operation. (3) No other lower limb diseases and no history of trauma after operation.

According to the inclusion criteria, a total of 197 patients were identified and invited to the outpatient department for the follow-up in June 2021. Thirty-nine patients were lost to follow-up, and the follow-up rate was 80.2%. Among the 158 patients available for analysis, standard MP insert was used in 84 patients, and DH insert in 74 patients.

For all patients undergoing joint replacement at our institution, preoperative functional outcomes were routinely recorded, including the range of motion (ROM), the Knee Society Score (KSS), the Knee Society Function Score (KSFS), and the Western Ontario and McMaster Universities Arthritis Index (WOMAC) score. Standing anteroposterior and lateral knee radiographs, as well as a weight-bearing full-length radiograph, were taken. Senior surgeons in our institution performed all TKA procedures, and a pneumatic tourniquet was applied throughout the procedure. A medial parapatellar approach was used during the operation to expose the knee, and the femoral osteotomy was performed through mechanical alignment technique and 3° of external rotation. An extramedullary guide was used to cut the proximal tibia after protecting the tibial insertion of the posterior cruciate ligament (PCL). After patella resurfaced, all components were fixed with bone cement. Either DH or MP inserts were used. Finally, a drainage tube was placed, and the incision was closed. The routine postoperative care includes intravenous cefuroxime administration to prevent infection, subcutaneous low-molecular-weight heparin to prevent venous thromboembolism (VTE), intravenous non-steroid anti-inflammatory drugs (NSAIDs) followed by oral administration, and daily continuous passive motion.

The postoperative follow-up included a routine visit to the outpatient department at 6 weeks and 1 year postoperatively, as well as this final follow-up. Senior residents conducted all physical examinations and evaluated the clinical outcomes using the ROM, the KSS, the KSFS, and the WOMAC score. Self-reported outcomes were also evaluated using the Forgotten Joint Score (FJS). Radiographic results were read on a picture archiving and communication system (General Electric, Chicago, IL, USA) and measured using a mouse-point cursor and an automated computer calculator. Anteroposterior and lateral images of operated knee and standing anteroposterior images of lower limb at last follow-up were recorded. The femorotibial angle (FTA), hip-knee-ankle (HKA) angle and coronal position of the components, including anatomical medial proximal tibial angle (aMPTA) and anatomical lateral distal femoral angle (aLDFA), were measured. The posterior tibial slope (PTS) was also measured. The presence and location of radiolucent lines were also identified. All measurements were done using the standard method reported by Park et al. [14] and Kim et al. [15]. A radiolucency less than 1 mm was considered a physiological radiolucent line. A pathological radiolucent line was determined as a complete radiolucency of more than 1 mm, indicating a possible implant loosening.

**Fig. 1:**
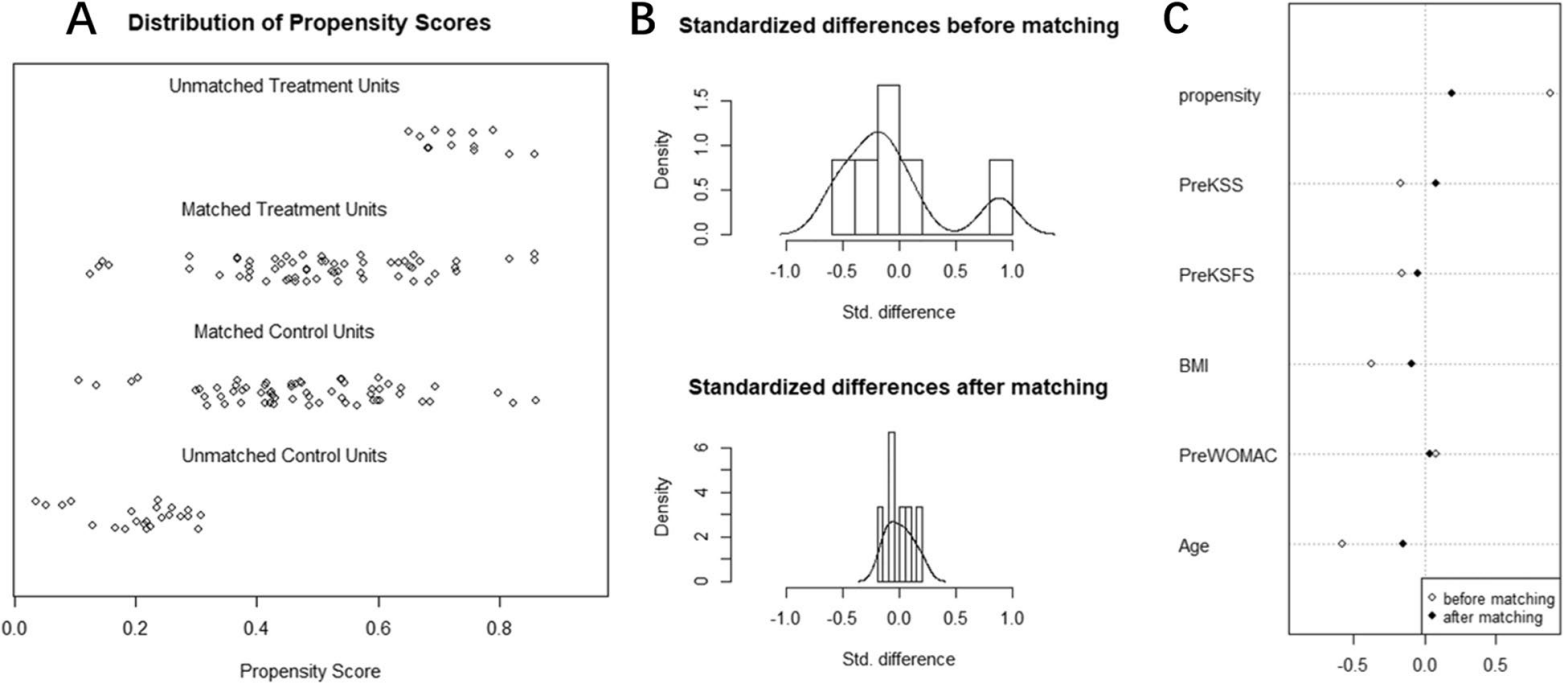
Results of propensity score matching. **A** Density plots after propensity score matching. **B** Standarized differences before and after propensity score matching. **C** Confounding variables before and after propensity score matching

A 1:1 propensity score-matched analysis was then performed between the patients with MP and DH inserts to control selection bias and ensure covariate balance between the groups. The confounding variables, including age, body mass index (BMI), and baseline clinical scores such as KSS, KSFS, and WOMAC score, were matched using propensity score calculated by logistic regression. The propensity scores and standardized differences before and after matching were calculated and shown in Fig. 1.

### Statistical analysis

Statistical analysis was done by Statistical Package for the Social Sciences 26.0 (SPSS Inc., Chicago, IL). Clinical data were presented as mean ± standard deviation (SD). The difference in continuous variables was compared using the student t-tests. Fisher exact test was used to determine the difference in categorical variables. *P* < 0.05 was considered statistically significant.

## Results

After a 1:1 propensity score matching, the data of 120 patients (60 pairs) were analyzed. There was no statistical difference in demographic data including age, BMI, and gender ratio between DH and MP groups. The length of stay and follow-up period between the DH and MP groups were also comparable (Table 1). Preoperatively, no significant difference was found in baseline WOMAC score (76.2 ± 4.7 vs. 75.9 ± 6.6, *p* = 0.740), KSS (23.4 ± 7.1 vs. 22.8 ± 4.8, *p* = 0.587), KSFS (31.9 ± 12.3 vs. 32.6 ± 14.4, *p* = 0.785), and ROM (82.5 ± 16.4 vs. 84.0 ± 12.9, *p* = 0.632, Table 2). In both groups, significantly improved postoperative clinical outcomes were found (Table 2). At the last follow-up, although the DH group ROM was significantly higher than the MP group’s (111.0 ± 10.5 vs. 104.3 ± 11.8, *p* = 0.001), the KSS (82.9 ± 11.7 vs. 90.2 ± 5.4, *p* = 0.000), WOMAC score (28.1 ± 9.6 vs. 11.6 ± 13.4, p = 0.000), and FJS (66.7 ± 3.6 vs. 77.1 ± 24.0, p = 0.000) were all in favor of MP group. The postoperative KSFS (73.7 ± 12.7 vs. 73.5 ± 12.3, *p* = 0.942) was found to be equivalent between the two groups (Table 2).

**Table 1:** Demographics information

Parameters	DH group	MP group	*P* value
Age (year)	65.4 ± 6.8	66.4 ± 7.2	0.421
Male (%)	20.0%	10.0%	0.132
BMI (kg/m^2^)	26.9 ± 3.6	27.2 ± 3.2	0.600
Follow-up (Range, Year)	8.6 ± 0.7 (7.0–10.2)	8.5 ± 1.1 (7.0–10.2)	0.812
Length of stay (LOS, Day)	10.7 ± 8.4	10.0 ± 3.5	0.603

**Table 2:** Mid-term clinical outcomes between PS group and CR group

Parameters	DH group	MP group	*P* value
Preoperative WOMAC	76.2 ± 4.7	75.9 ± 6.6	N.S.
Postoperative WOMAC	28.1 ± 9.6	11.6 ± 13.4	0.000
*P* value	0.000	0.000	
Preoperative KSS	23.4 ± 7.1	22.8 ± 4.8	N.S.
Postoperative KSS	82.9 ± 11.7	90.2 ± 5.4	0.000
*P* value	0.000	0.000	
Preoperative KSFS	31.9 ± 12.3	32.6 ± 14.4	N.S.
Postoperative KSFS	73.7 ± 12.7	73.5 ± 12.3	N.S.
*P* value	0.000	0.000	
Preoperative ROM	82.5 ± 16.4	84.0 ± 12.9	N.S.
Postoperative ROM	111.0 ± 10.5	104.3 ± 11.8	0.001
*P* value	0.000	0.000	
FJS	66.7 ± 3.6	77.1 ± 24.0	0.000

**Fig. 2:**
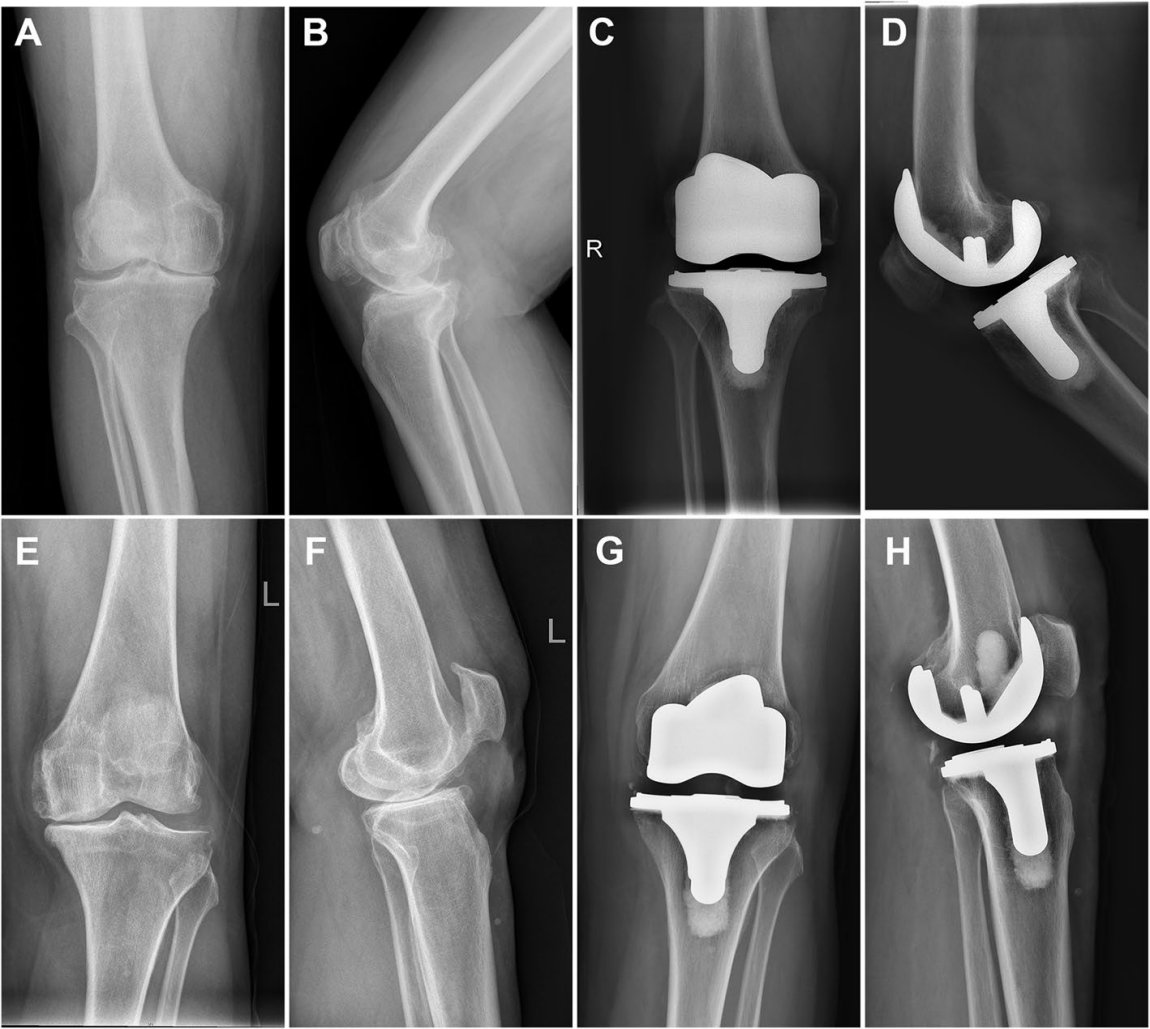
Preoperative and final X-ray of medial-pivotal (MP) insert (panel **A**-**D**) and double-high (DH) insert (panel **E**-**H**) used in cruciate-retaining TKA

The radiographic outcomes are displayed in Table 3 and Fig. 2. The preoperative FTA measured on anteroposterior image and the HKA measured on standing anteroposterior images of lower limb between DH and MP groups were not statistically different (FTA: − 4.3° ± 6.6 vs. -4.4° ± 7.2, *p* = 0.632; HKA: 10° ± 4.2° vs 9.7° ± 4.5°, *p* = 0.770). Postoperatively, the alignment of the lower limb was corrected in both groups. The FTA was 3.9° ± 2.7° and 3.7° ± 3.5° in DH and MP groups, respectively, without statistical significance (*p* = 0.589), and the HKA was 1.3° ± 2.4° and 1.5° ± 2.3° in two groups, respectively, without statistical significance (*p* = 0.712). The position of the femoral and tibial components in both groups was satisfactory, indicated by normal aLDFA (83.9° ± 2.2 vs. 84.7° ± 2.2, *p* = 0.100), aMPTA (88.3° ± 2.7 vs. 88.4° ± 2.3, *p* = 0.767), and PTS (4.0° ± 6.2 vs. 4.2° ± 5.8, *p* = 0.848). No pathological radiolucent line was found in both groups (Table 3).

**Table 3:** Radiographic Results

Parameters	DH group	MP group	*P* value
Femorotibial angle (degrees)			
Preoperative	−4.3 ± 6.6	−4.3 ± 7.2	N.S.
Final follow-up	3.9 ± 2.7	3.7 ± 3.5	N.S.
Hip-Knee-ankle angle (degrees)			
Preoperative	10 ± 4.2	9.7 ± 4.5	N.S.
Final follow-up	1.3 ± 2.4	1.5 ± 2.3	N.S.
aLDFA (degrees)	83.9 ± 2.2	84.7 ± 2.2	N.S.
aMPTA (degrees)	88.3 ± 2.7	88.4 ± 2.3	N.S.
Posterior tibial slope (degrees)	4.0 ± 6.2	4.2 ± 5.8	N.S.
Radiolucent line ≤1 mm	4 (6.7%)	3 (5.0%)	N.S.
Radiolucent line > 1 mm	0	0	N.S.

Among the 60 pairs propensity matched patients, seven complications were recorded. There were three complications in the MP group (5%), including one case of continuous patellar clicking and two cases of anterior knee pain. Four complications were found in the DH group (6.7%), including one case of continuous patellar clicking, two cases of anterior knee pain and one case of periprosthetic infection. The patient suffering periprosthetic infection underwent revision TKA and none of the other patients underwent reoperation. The complication and revision rates between the two groups lacked statistical significance (Table 4).

**Table 4:** Overall complications

Complications	DH group (*n* = 60)	MP group (*n* = 60)	*P* value
Infection	1		
Complications of patellofemoral joint			
Continuous patellar clicking	1	1	
Anterior knee pain	2	2	
**Total**	4 (6.7%)	3 (5.0%)	1.00
**Revisions**	1 (1.7%)	0	1.00

## Discussion

The ADVANCE® knee system is one of the most widely applied medial-pivot (MP) knee implants worldwide which is characterized by the conformed medial ball-socket articulating interface [16]. This unique design diminishes the paradoxical anterior translation of the femur, and good to excellent long-term outcomes and implant survivorship have been reported [17]. Two types of the tibial insert have been designed for ADVANCE® knee and can be exchanged on the same tibial tray. Compared to the conventional MP insert, the anterior and posterior lips of the DH insert are decreased by 1 mm and 3 mm, respectively, forming a path for femoral rollback and aiming to achieve better flexion. In this study, the postoperative clinical outcomes of both groups were significantly improved. And a better ROM was found in DH group than MP group, but in KSS, WOMAC and FJS, the MP group was superior to the DH group.

DH insert is not widely used in clinic and investigation on the clinical and radiographic outcomes of DH insert is rare. There has only been one published research investigating its clinical application. A comparison between MP and DH inserts was conducted by Ishida et al. in cruciate-sacrificing TKA, including 20 knees in each group. After a mean 4-year follow-up, they found that the KSS, KSFS, and University of California, Los Angeles (UCLA) activity scores between the DH and MP groups were not significantly different. However, the KSS of the MP group was four points higher. Meanwhile, the DH group presented better ROM and knee flexion, but significance was still not found [18].

The main differences between Ishida’s research and this are the mean follow-up (4 years vs 8 years,), surgical methods (cruciate-sacrificing TKA vs cruciate-retaining TKA), research methods (prospective randomized controlled trial vs retrospective propensity score-matched analysis), sample size (40 vs 120) and some of the research results, as above. One of the reasons for the different results may be that this study have a larger sample size and longer follow-up. The more important reason for the difference in ROM comes from the different treatment of PCL. Resecting PCL allows a larger flexion-gap and facilitates the femoral rollback cand flexion motion in the MP inserts. So the DH inserts did not show an advantage in ROM in cruciate-sacrificing TKA. Therefore the DH inserts are more suitable for cruciate-retaining TKA than cruciate-sacrificing TKA.

To some extent, this results partly agreed with the results of Ishida et al. Comparable KSFS and radiographic results between the two groups, superior KSS of MP group and better ROM of DH group were observed. However, compared with that of the DH group, the KSS of the MP group was seven points higher, and the ROM was 10° worse, both with statistical significance.

The FTA of the DH and MP groups was averaged 3.9° and 3.7°, and the HKA was averaged 1.3° and 1.5°, respectively. According to previous studies, the alignment was considered varus when the FTA was less than 2.4°-4° [15, 19, 20], and the alignment was considered neutral when the HKA was in the range of − 3° to 3° [14]. The results indicated that both groups had good alignment neutrality, which is important to implant survivorship. The coronal and the sagittal positions of the implant were also measured, and the results showed that the aLDFA, aMPTA, and PTS were all equally good between the two groups. With the similar PTS, a 3 mm reduction in posterior lip of DH insert increases the flexion gap. Thus, the better ROM of the DH group is reasonable. When referring to significantly worse KSS, WOMAC score, and FJS of DH group, it is reasonable to question whether this increased posterior slope of DH insert might cause knee instability. However, no patient complained of unstable knee as a complication. In a previous research about the suitability of the DH insert for the cruciate-retaining procedure, Omori et al. specifically investigated the influence of different geometry of DH and MP insert on knee kinematics [13]. According to their results, a similar motion pattern was observed between MP and DH inserts under cruciate-retaining conditions. Both inserts failed to reproduce medial-pivotal motion but did have bicondylar femoral rollback. However, under the cruciate-sacrificing condition, although medial-pivotal kinematics were confirmed, paradoxical anterior translation of the lateral compartment was also observed from 0° to 60° of knee flexion, indicating that DH insert might not be suitable for cruciate-sacrificing procedures. Thus, There may be some minor problems in the DH design, leading to worse mid-term clinical outcomes than the MP insert. And this might be the reason why DH insert is not widely used. This study also reported that the mid-term clinical outcomes of DH insert in this study are relatively good for most patients, but some modifications are needed to get better results.

This study provided the first report on comparing clinical outcomes between DH insert and MP inserts in cruciate-retaining ADVANCE® TKA with the longest follow-up period, and the largest number of patients included. However, this study had several limitations. Firstly, the follow-up time and sample size of this study may not be enough for the verification of some of the research results. And this was a single-center study which might compromise the generalizability. Secondly, although we attempt to avoid bias through propensity score matching, the retrospective design of this study inevitably led to bias during data analysis. However, since 2015, the DH insert was unavailable, and the cruciate-sacrificing procedure was predominantly performed using the MP insert in our institution. Thus, we could not carry out a prospective study. Prospective RCT with larger sample size and longer follow-up are needed in the future.

## Conclusion

In this averaged 8-year follow-up, both MP and DH inserts presented good mid-term clinical outcomes in cruciate-retaining TKA, with low complication and revision rates. However, we found MP insert was superior to DH insert in clinical outcomes through a propensity score matching analysis, including KSS, WOMAC score, and FJS.

## Data Availability

None.

## Abbreviations

MP: Medial-pivotal
DH: Double-high
TKA: Total knee arthroplasty
PSM: Propensity score matching
ROM: Range of motion
KSFS: Knee Society Function Score
KSS: Knee Society Score
WOMAC: Western Ontario and McMaster Universities Arthritis Index
FJS: Forgotten Joint Score
PCL: Posterior cruciate ligament
VTE: Venous thromboembolism
NSAIDs: Non-steroid anti-inflammatory drugs
FTA: Femorotibial angle
aMPTA: Anatomical medial proximal tibial angle
aLDFA: Anatomical lateral distal femoral angle
PTS: Posterior tibial slope
